# Optimal Design and Operation of an Ultrasonic Driving System for Algae Removal Considering Underwater Environment Load

**DOI:** 10.3390/s25020542

**Published:** 2025-01-18

**Authors:** Changdae Joo, Taekue Kim

**Affiliations:** Department of Electric Engineering, Changwon National University, Changwon 51140, Republic of Korea; ckd422@naver.com

**Keywords:** piezoelectric transducer, application of ultrasonic vibration, algae removal system, resonant VSI, finite element analysis

## Abstract

This study investigates the optimal design and operation of an underwater ultrasonic system for algae removal, focusing on the electromechanical load of Langevin-type piezoelectric transducers. These piezoelectric transducers, which operate in underwater environments, exhibit variations in electrical–mechanical impedance due to practical environmental factors, such as waterproof molding structures or variations in pressure and flow rates depending on the water depth. To address these challenges, we modeled the underwater load conditions using the finite element method and analyzed the impedance characteristics of the piezoelectric transducer under realistic environmental conditions. Based on this analysis, we developed an ultrasound-driven system capable of efficient output control by incorporating the impedance characteristics of the transducer under load variations and subaquatic conditions. This study proposes analytical and experimental methods for modeling and analyzing practical ultrasound-driven systems for algae removal.

## 1. Introduction

**Figure 1:**
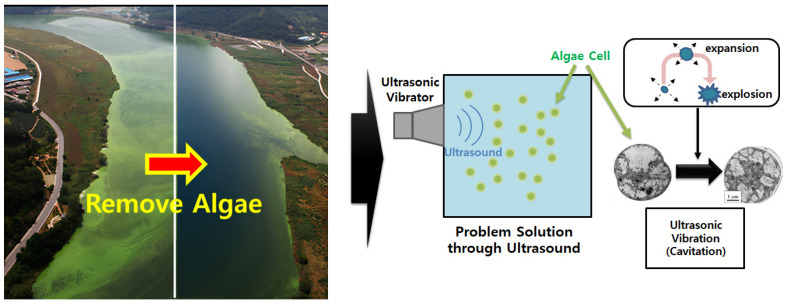
Environmental problems caused by algae and air sac destruction in cells via ultrasonic action.

The increasing severity of environmental issues, such as global warming and the increased occurrence of algae blooms that degrade water quality, has become a pressing concern. Ultrasonic technology has emerged as a promising approach for controlling algae growth and improving aquatic environments, as in Figure 1 [1,2,3]. This study employs a Langevin-type piezoelectric transducer for underwater ultrasonic applications. In such applications, the piezoelectric transducer must operate underwater, where environmental factors, such as pressure and flow variations caused by water depth, induce mechanical loads. These loads alter the electromechanical impedance of the piezoelectric transducer. Furthermore, impedance mismatch occurs when the transducer operates with simple frequency control, reducing its power conversion efficiency [4,5].

This paper models the underwater load conditions and waterproof molding structures required for the installation of a piezoelectric transducer and analyzes the impedance characteristics using the finite element method (FEM). Based on these impedance characteristics, an optimal model was designed to minimize the reduction in the mechanical quality factor. Additionally, variations in the load and impedance of the piezoelectric transducer caused by high-power operation and underwater environmental factors were pre-analyzed to design an ultrasonic driving circuit [4,5,6,7,8,9].

**Figure 2:**
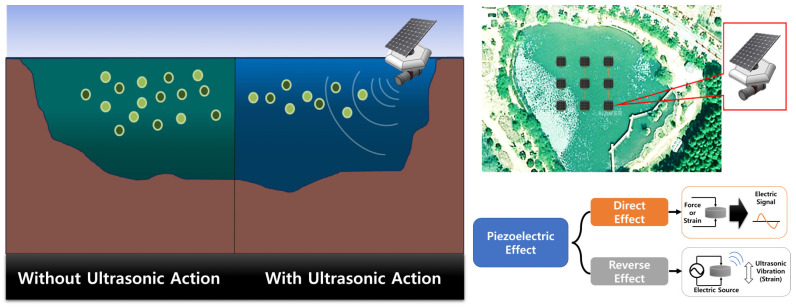
Ultrasonic action in the proposed algae removal system.

As previously mentioned, the frequency of algae occurrences has been increasing globally due to environmental issues. Consequently, various studies have explored methods to suppress algae growth and improve aquatic environments, with ultrasonic action emerging as a potential solution [1,2,3,10,11,12,13,14]. To address this issue, this study proposes an algae removal system using ultrasonic vibrations, which is designed as a grid system capable of covering large areas, such as lakes and rivers, as in Figure 2.

The transducer’s reverse piezoelectric effect must be applied to employ ultrasonic vibrations to suppress algae blooms, necessitating a power conversion device capable of controlling the input power. An LLC resonant tank was designed to secure the operating range based on the resonant frequency of the Langevin piezoelectric transducer. Additionally, impedance matching can be improved through inductive compensation, considering the capacitive characteristics of the piezoelectric transducer [4].

To maximize the output driving of the piezoelectric transducer, the transfer functions of the voltage and current applied to the transducer were derived, and the frequency characteristics of both were analyzed. To achieve maximum active power, the system must operate in a range where the voltage gain is maximized, and the voltage and current are in-phase. Therefore, the optimal operation was verified near the resonance point by analyzing the frequency characteristics of the voltage and current of the piezoelectric transducer. The Langevin-type piezoelectric transducer used in this study was based on previous research (C.D.Joo, 2022) results, including free-body (unloaded) conditions, underwater load modeling, and FEM impedance characteristic analysis [15]. Overall, this study focused on the optimal modeling and design of a practical case of an ultrasonic driving system for algae removal based on both analytical and experimental data.

## 2. Methodology and Approach

### 2.1. Impedance Characteristics Analysis in Underwater Load Modeling

**Figure 3:**
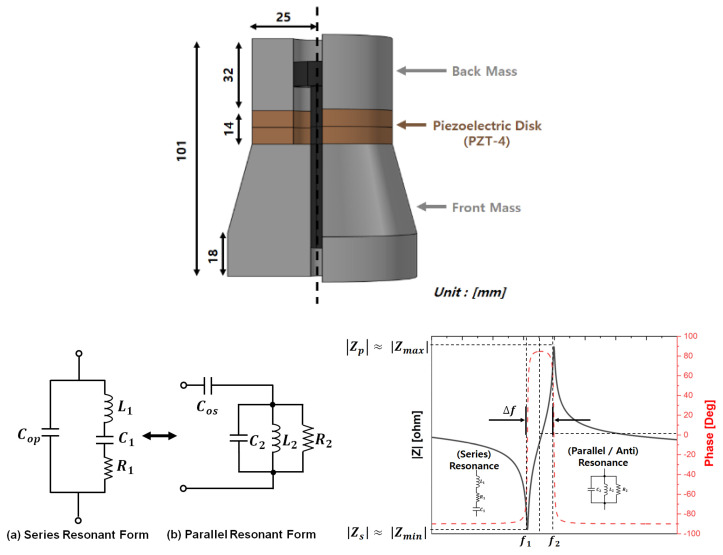
Shape of the Langevin-type transducer and BVD-equivalent circuit model.

In typical ultrasonic applications, such as ultrasonic welding, single transducers operating at a single frequency are commonly used. In contrast, this study employed a bolted-clamped Langevin transducer with multiple resonant frequencies to target various algae cell types. The configuration of the transducer used in this study and the BVD (Butterworth–Van Dyke)-equivalent circuit [4,15] used for circuit analysis are shown in Figure 3.

In this study, we conducted FEM simulations of the piezoelectric effect based on the IEEE 1978 standard [9]. The strain-charge form of constitutive relations was used for the multiphysics analysis of the piezoelectric transducer, accounting for the piezoelectric effect and acoustic pressure. Equations (1) and (2) represent the governing of electromechanical equations.

(1) $$\vec{S}=s_{E}\vec{T}+d^{T}\vec{E}$$

(2) $$\vec{D}=d\vec{T}+\varepsilon_{0}\varepsilon_{rT}\vec{E},$$

where $\vec{S}$ is the strain, $\vec{T}$ is the stress, $\vec{E}$ is the electrical field, $\vec{D}$ is the electric displacement field, $s_{E}$ is the material compliance, *d* is the coupling properties, $\varepsilon_{0}$ is the permittivity of free space, and $\varepsilon_{rT}$ is the relative permittivity at constant stress.

A piezoelectric transducer functions as a converter between electrical and mechanical energy, and its energy conversion efficiency increases with the mechanical quality factor. The mechanical quality factor ($Q_{m}$), a key performance indicator for the mechanical response characteristics of a piezoelectric transducer, quantifies the efficiency with which the electrical energy applied to the transducer is converted into mechanical response. The mechanical quality factor ($Q_{m}$) and mechanical damping coefficient (ζ) of the piezoelectric transducer in the free-body state can be calculated as shown in Equations (3) and (4).

(3) $$Q_{m}=\frac{Z_{m}}{R_{1}}=\frac{1}{R_{1}}\sqrt{\frac{L_{1}}{C_{1}}}\;≅866.09$$

(4) $$\zeta \;≅\;\frac{1}{2Q_{m}}=0.000577$$

**Figure 4:**
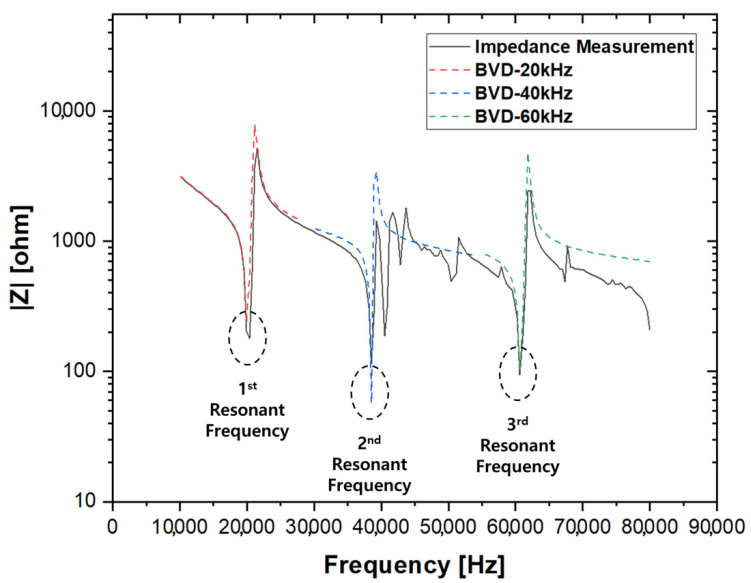
Comparison of measured impedance and impedance obtained from BVD modeling.

Several considerations and limitations must be addressed when integrating the piezoelectric transducer into underwater ultrasonic driving systems. First, the Butterworth–Van Dyke-equivalent circuit model, which serves as the basis for the design of the transducer’s electrical driving circuit, effectively reflects the impedance characteristics near the resonant frequency, as shown in Figure 4. However, it does not accurately account for impedance beyond each resonant point. Second, the transducer requires a waterproof structure for underwater operation because water pressure imposes a mechanical load on the transducer, significantly reducing the mechanical quality factor, as indicated by the FEM analysis. Furthermore, to ensure electrical safety and improve the mechanical response of the transducer, designing a sealed and enclosed module for the transducer is crucial.

In this study, the impedance characteristics, which serve as the key electrical parameters for driving the ultrasonic transducer, were derived by FEM analysis. When designing based on the BVD-equivalent circuit of a piezoelectric transducer, simple resistive load modeling is insufficient to account for the changes in the resonant frequency caused by impedance variations. To develop an underwater ultrasonic driving system for suppressing algae growth and to integrate a Langevin-type piezoelectric transducer, previous research [15] was used to model underwater loads and analyze the impedance characteristics.

**Figure 5:**
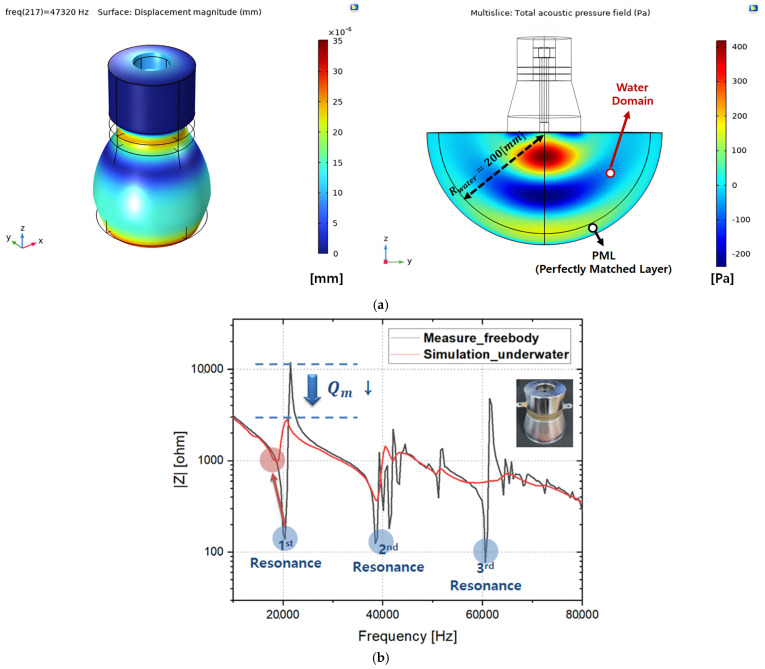
(**a**) FEM multiphysics analysis results in COMSOL and (**b**) impedance characteristics curve of COMSOL (underwater) and measurement (free body).

An FEM impedance analysis was conducted for the Langevin-type transducer under underwater load conditions, and the simulation results for the transducer fully submerged in water were compared with the impedance measurements in a free-body state, as shown in Figure 5 [6,8,15]. Based on the previous study [15], an FEM-based Piezoelectric Effect and Acoustic-Structure multiphysics analysis were performed to model the underwater load conditions for the piezoelectric transducer. A water domain surrounded by a PML (Perfectly Matched Layer) physical boundary condition was designed to apply ultrasonic waves and pressure propagating underwater according to the acoustic analysis. Meanwhile, for the water domain designed as a hemisphere with a radius of approximately 200 [mm], physical boundary conditions (Physics) were designed through the Infinite Element Domain so that it can be effectively applied to a very long distance beyond the boundary conditions of the actual geometry domain.

As shown in Figure 5b, when the transducer operates underwater without a waterproof structure, the water pressure acts as a mechanical load, significantly reducing the mechanical quality factor ($Q_{m}$). To ensure electrical safety and improve the mechanical response characteristics of the piezoelectric transducer at various water depths, the transducer was enclosed and sealed within a protective structure. However, when the enclosure is filled with epoxy or similar materials, the displacement of the transducer decreases. Therefore, the enclosure near the front mass area must be secured while ensuring that the piezo disk remains as close to a free-body state as possible.

### 2.2. Prototype Module Design and FEM Analysis Results

**Figure 6:**
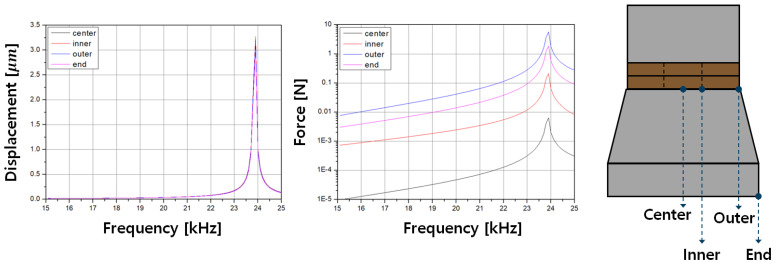
Transducer’s displacement and force through FEM analysis results.

This study aimed to design an optimal model to minimize the reduction in the mechanical quality factor when employing ultrasonic transducers in underwater ultrasonic driving systems. The critical design considerations for the sealing and casing of the transducer module are the force and displacement. To evaluate performance, the displacement and force generated by the reverse piezoelectric effect in the free-body state of the piezoelectric transducer were used as benchmarks, as shown in Figure 6. The objective was to ensure that enclosing the transducer for underwater operation and safety does not degrade its vibration performance. The simulation models were evaluated based on four key points of the transducer: Center, Inner, Outer, and End [15,16].

Finite element method (FEM) analyses were conducted to evaluate various cases by comparing housing materials (plastic and aluminum) and the degree of fixation.

**Figure 7:**
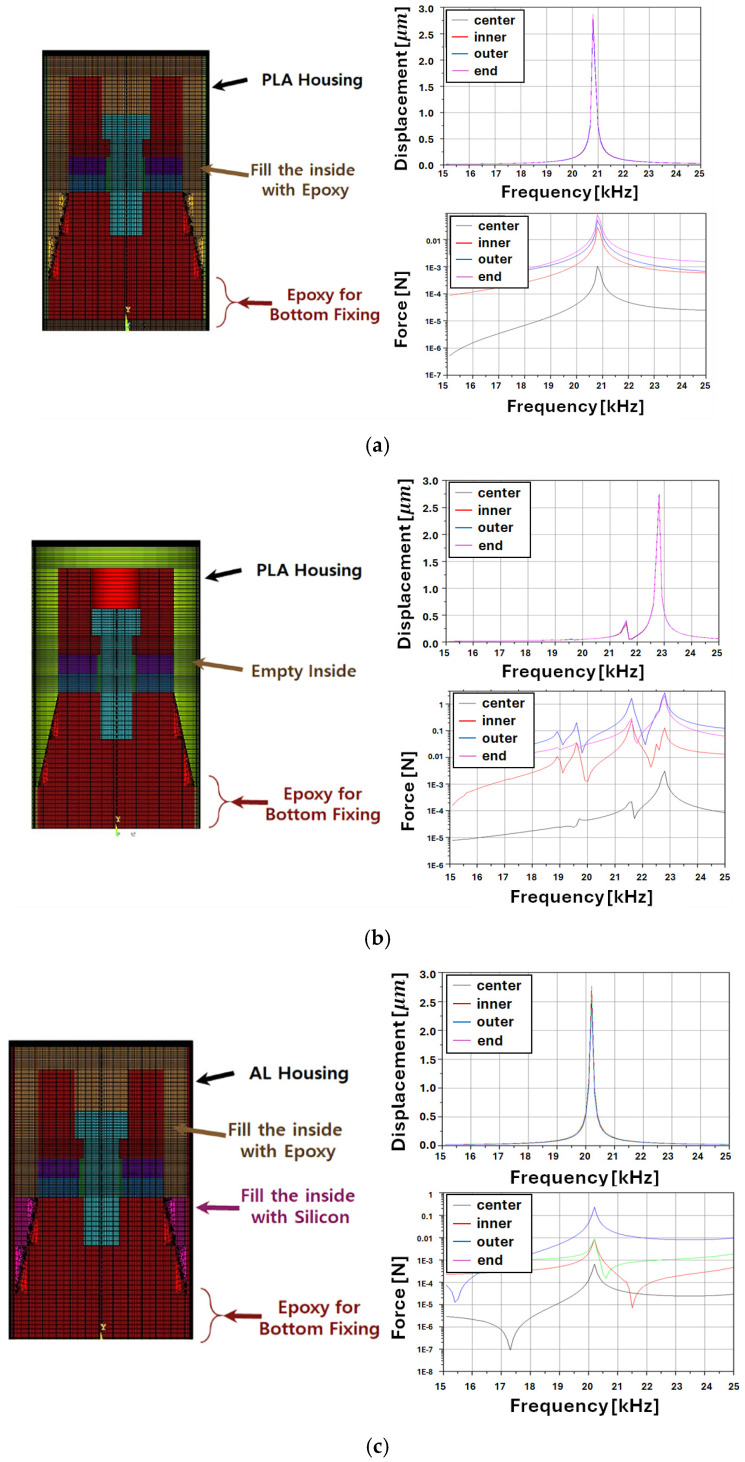

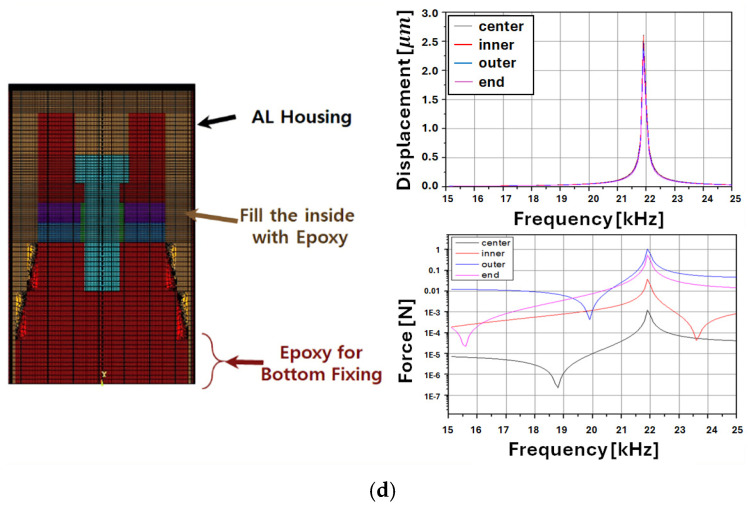
Simulation results of displacement and force based on transducer module design under various conditions: (**a**) PLA housing, epoxy-filled; (**b**) PLA housing, empty; (**c**) AL housing, epoxy + silicon-filled; and (**d**) AL housing, epoxy-filled.

During the initial prototype design stage, the housing was intended to be fabricated using a 3D printer. The FEM analysis results for these cases are shown in Figure 7a,b. Aluminum was selected as the housing material to improve the vibration transmission and strain compared with PLA, as depicted in Figure 7c,d. Additionally, to ensure the waterproof stability of the transducer, the FEM analysis was conducted with the housing interior filled with epoxy or silicone. However, this structure introduced an additional mechanical load on the transducer, which increased the mechanical damping.

**Figure 8:**
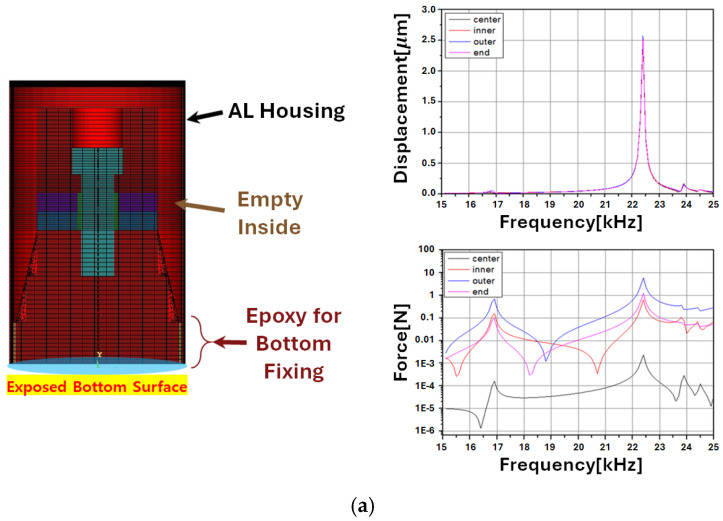

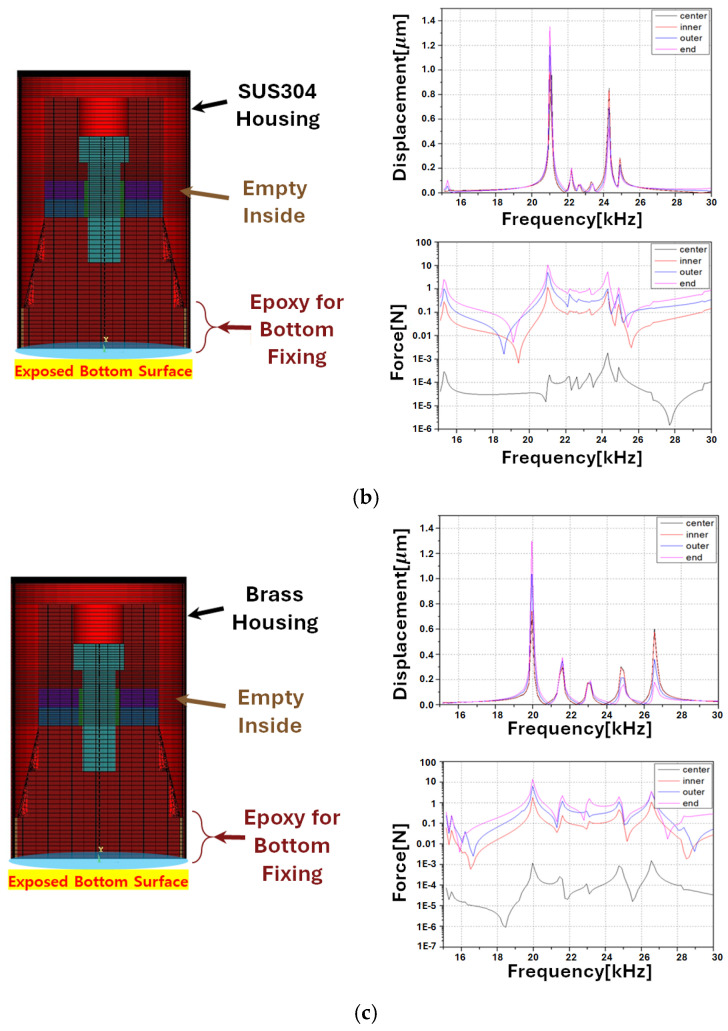
Comparative analysis of prototype optimal design based on module housing material: (**a**) AL housing; (**b**) SUS304 housing; and (**c**) Brass housing.

Based on the analysis results of various transducer module designs, the optimal model was identified, which exhibited superior performance in terms of force and displacement. To mitigate the reduction in displacement caused by epoxy filling, only the front mass area was fixed. Additionally, the design ensured that the piezoelectric disk remained as close to a free-body state as possible. As shown in Figure 8a, the simulation results confirm that the vibration displacement and force values closely match the performance of the free-body transducer shown in Figure 6.

Additionally, based on the FEM analysis results of the transducer module design described earlier, Figure 8 presents the simulation results for the force and displacement corresponding to changes in the housing material (AL/SUS304/Brass). These results are derived from the conclusion that leaving the area around the piezo disk in a free state while partially fixing only the bottom of the module for the transducer’s fixation yields higher force and displacement.

Therefore, the key comparison in Figure 8 focuses on the performance indicators of the transducer module—force and displacement—depending on the housing material. These comparisons were conducted using the FEM analysis in the ANSYS Workbench environment. Additionally, quantitative indicators for these analysis results are summarized in Table 1.

Based on the content of Table 1, the region showing the highest force for each module design case was either the Outer or End region. Under the conditions of Figure 8a, the results for the Outer region were most similar between the free-state condition and the final module condition, both showing approximately 5.8 N. Additionally, the FEM analysis ranked the magnitude of force as Outer > End > Inner > Center, while displacement performance remained relatively consistent across all the regions.

Furthermore, when comparing the average displacement in each region for modules designed with different housing materials, the ranking was AL > SUS304 > Brass. For the average force, the ranking was Brass > SUS304 > AL. In other words, considering only displacement, AL demonstrated the highest values across all the regions, while Brass exhibited the highest force.

**Figure 9:**
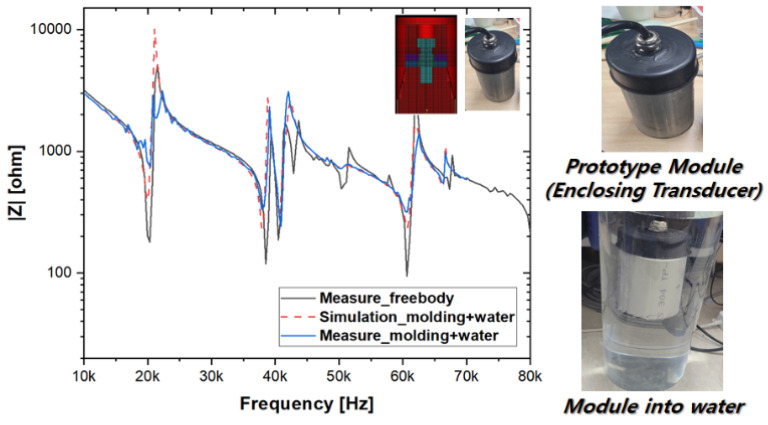
Prototype module design and impedance analysis results: comparison of measurements with simulations results.

However, in this study, considering both results comprehensively, SUS304, which showed balanced and normal performance across all the regions, was selected as the housing material for the final prototype. Subsequently, as shown in Figure 9, a prototype module was developed, and the electrical characteristics of the ultrasonic application were verified by comparing the FEM simulation results with actual impedance measurements [6,8,15].

## 3. Driving Circuit and System Design

### Optimal Design of the Ultrasonic Driving Circuit System for Algae Removal Application

**Figure 10:**
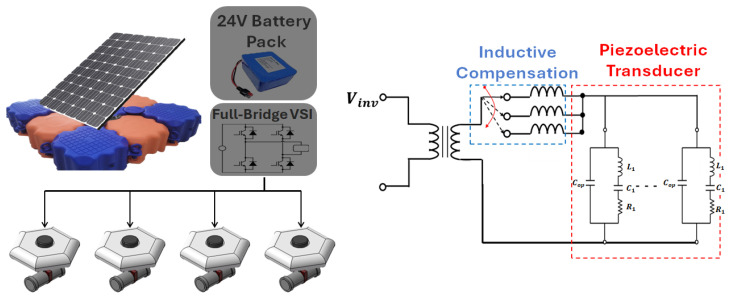
Concept of the ultrasonic driving system for algae removal.

To implement the grid-type system described above, each module was designed as a small system. The primary DC link source comprised a 1 kWh PV panel and a 24 V battery pack. A full-bridge voltage source inverter supplied power to the transducer in four directions (Figure 10). In the algae removal system, the transducers operate at multiple resonances—20, 40, and 60 kHz—to enhance the efficiency of algae removal. This functionality is achieved using relays to adjust the inductive compensation, ensuring that the piezoelectric transducer receives the appropriate AC voltage and current at each resonance point.

Applying an electrical input matched to its mechanical resonant frequency is essential to achieve optimal driving of the piezoelectric transducer. Therefore, a voltage source inverter (VSI)-based resonant inverter was designed [5]. The resonant frequency of all resonant circuits was determined based on the mechanical resonant frequency of the piezoelectric transducer, which corresponds to the frequency at which its impedance is minimized.

**Figure 11:**
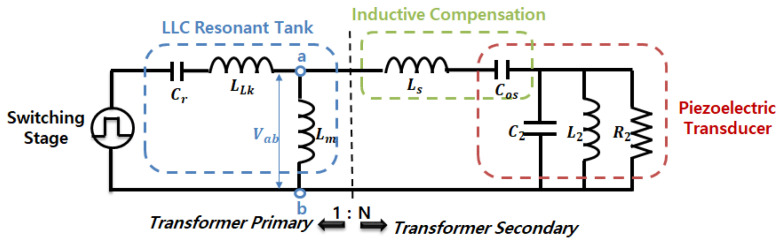
Configuration of the VSI resonant circuit.

Based on the equivalent circuit of the ultrasonic driver circuit shown in Figure 11, the mechanical resonant frequency ($f_{r1}$) of the piezoelectric transducer and the resonant frequency ($f_{r2}$) of the LLC resonant tank can be calculated using Equations (5) and (6), respectively. Additionally, to generate a sine-wave input voltage at the mechanical resonant frequency from the square-wave pole voltage applied by the full-bridge switching circuit, the resonant tank was designed to satisfy the condition ($f_{r1}=f_{r2}$).

The resonant frequency of the piezoelectric transducer ($f_{r1}$) is expressed as follows:

(5) $$f_{r1}=\frac{1}{2\pi \sqrt{L_{2}C_{2}}}$$

The resonant frequency of the LLC resonant tank ($f_{r2}$) is expressed as follows:

(6) $$f_{r2}=\frac{1}{2\pi \sqrt{{(L}_{lk}+L_{m})C_{r}}}$$

where $L_{2}$ and $C_{2}$ in Equation (5) represent the mechanical resonance components that determine the parallel resonant frequency or anti-resonant frequency, based on the parallel mode of the BVD-equivalent circuit model illustrated in Figure 3 [4]. These components are derived using the parameters for the series resonance mode, such as $C_{op}$, $R_{1}$, $L_{1}$, and $C_{1}$, as specified in Table 2. The derivations are provided in Equations (7)–(10) below [4,15].

(7) $$C_{os}\;≅\;C_{op}+C_{1}$$

(8) $$C_{2}=(C_{1}+C_{op})\frac{f_{1}^{2}}{f_{2}^{2}-f_{1}^{2}}$$

(9) $$L_{2}=\frac{L_{1}C_{1}}{C_{os}}(1-\frac{C_{op}}{C_{os}})$$

(10) $$R_{2}\;≅|Z_{max}|$$

**Figure 12:**
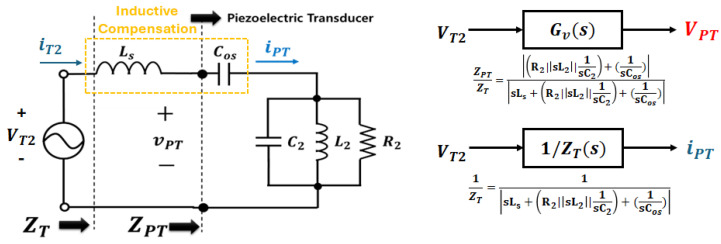
AC-equivalent analysis with PT and series inductor and the System Transfer Function (s-form).

The optimal driving and power transfer strategies addressed in this study aim to maximize the active power by operating at the transducer’s resonant frequency, where the voltage and current in the VSI are in-phase. This approach maximizes the active power under pure resistive load conditions. To achieve this, impedance matching was implemented using series inductive compensation, as shown in Figure 12 and Equation (11) [17,18,19,20,21,22]. This was supplemented by designing a series inductance to compensate for the flow of current because the impedance characteristics of the transducer become capacitive as the mechanical load on the piezoelectric transducer increases.

The optimal design conditions for the series inductance are as follows:

(11) $$\frac{1}{4\pi^{2}f_{2}^{2}C_{o}}<L_{s}\;\approx \frac{1}{4\pi^{2}f_{2}^{2}C_{0}\left(1-\frac{∆f}{2f_{2}}\right)}\;\approx \frac{1}{4\pi^{2}f_{1}f_{2}C_{0}}=4.3[mH]<\frac{1}{4\pi^{2}f_{1}^{2}C_{0}}$$

**Figure 13:**
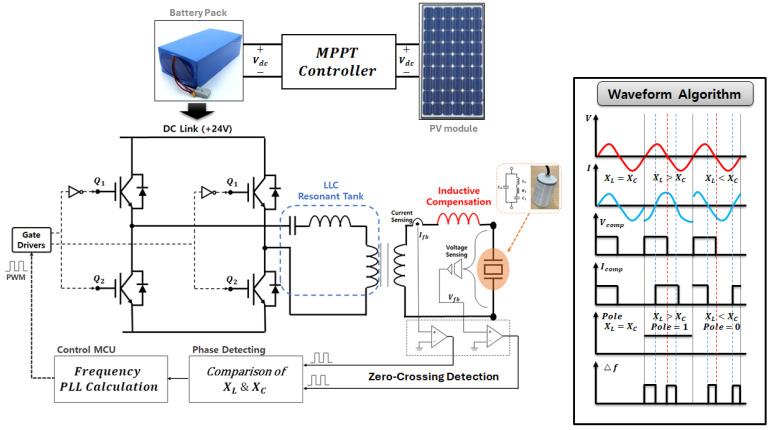
Configuration of the power converter control system and waveform algorithm.

The piezoelectric transducer employed in this study was integrated into an ultrasonic system that generates mechanical responses underwater using the reverse piezoelectric effect. Therefore, a power conversion device capable of controlling the input power was required, and the overall control and circuit topology are illustrated in Figure 13.

Additionally, to achieve maximum active power control, a PLL control system was configured using zero-crossing detection of the voltage and current of the piezoelectric transducer. The RLC parameters of the BVD-equivalent modeling used for circuit analysis and design are presented in Table 2 [17,18,19,20].

## 4. Experimental Results

### Design and Experimental Results of the Ultrasonic Application Hardware

**Figure 14:**
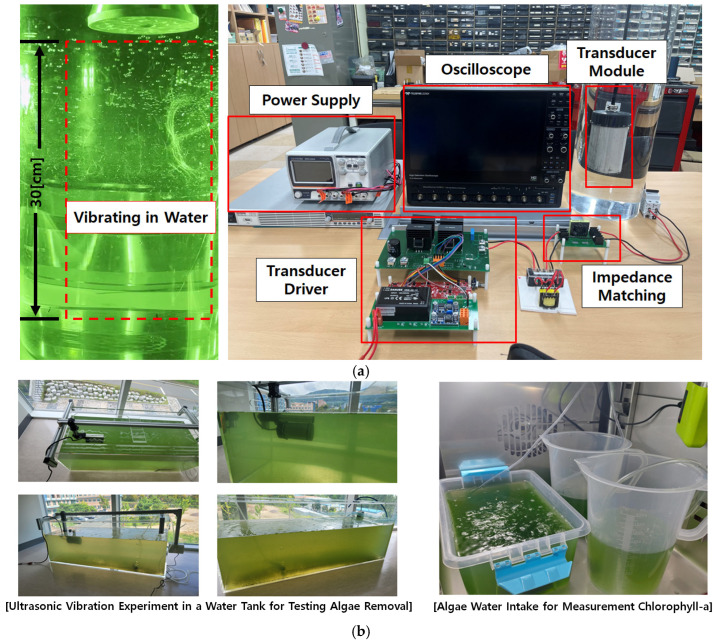
(**a**) Experimental, hardware configuration, and experiment result of transducer vibrating in water; (**b**) ultrasonic vibration experiment in water tank and algae water intake for measurement chlorophyll-a.

Figure 14 shows the overall experimental setup and cavitation phenomenon induced by ultrasonic driving underwater. Additionally, the water tank shown in Figure 14b is a tank with a length of approximately 1 [m], and the chlorophyll-a level was measured based on the microscopic counting method to verify the effectiveness of green algae removal [10,11,12,13,14,23,24].

**Figure 15:**
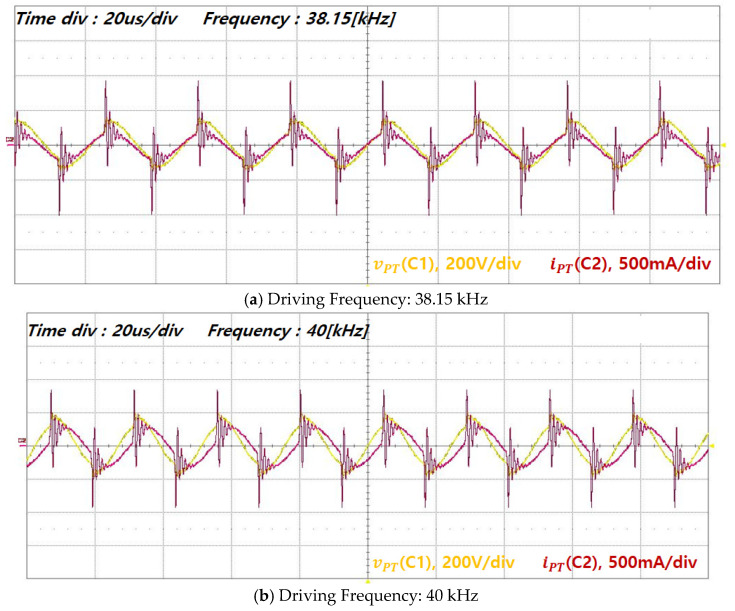
Transducer voltage and current without impedance matching: (**a**) 38.15 kHz and (**b**) 40 kHz.

**Figure 16:**
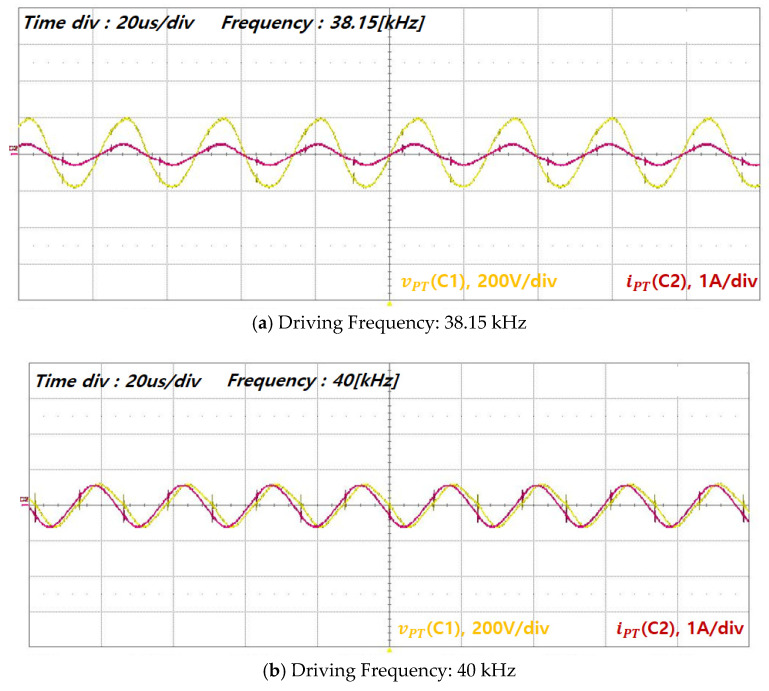
Transducer voltage and current with impedance matching: (**a**) 38.15 kHz and (**b**) 40 kHz.

The experimental waveforms of underwater ultrasonic operation obtained using the driving driver developed based on the previously described simulation and analysis results are presented. Figure 15 and Figure 16 compare the voltage and current waveforms of the piezoelectric transducer with and without series inductive compensation. The experimental results demonstrated that neither cavitation nor vibration occurred underwater without inductive compensation, and the current waveforms exhibited significant distortion. However, the driving driver was designed to ensure proper impedance matching, enabling the voltage and current waveforms of the piezoelectric transducer to be sinusoidal.

Consequently, the mechanical response of the transducer was significantly enhanced by applying a sinusoidal current output from the VSI through impedance matching.

An impedance characteristic analysis was performed at multiple resonance points to validate the transducer module design. The second resonance point (38.2 kHz and 40 kHz) was selected as the reference point due to its minimum impedance and relatively high $Q_{m}$. The corresponding voltage, current, and input power of the transducer at this resonance point are summarized in Table 3.

The experimental results for the transducer’s voltage ($v_{PT}$) and current ($i_{PT}$) near the second resonance point were consolidated into a graph as a function of the driving frequency. Piezoelectric transducers typically exhibit two resonance modes, resonance and anti-resonance, which are also referred to as series and parallel resonance modes, respectively. In the series resonance mode, the impedance is minimized, resulting in an increase in the current flowing into the piezoelectric transducer from the VSI. When an identical output power is applied to the transducer in both modes, the transducer operating in the series resonance state delivers a higher output force and a lower load speed. Conversely, the transducer operates at a higher speed with a relatively lower force in the parallel resonance mode. This behavior is based on the principle that the transducer’s output power is the product of the velocity and force transmitted to the emitting surface [4].

Therefore, to address high mechanical loads underwater and to ensure the effective transmission of acoustic waves and vibrations, the module was designed to minimize the reduction in force observed in the pure transducer state. The system operates in the series resonance mode, which facilitates the flow of higher electrical currents into the transducer [4,9].

**Figure 17:**
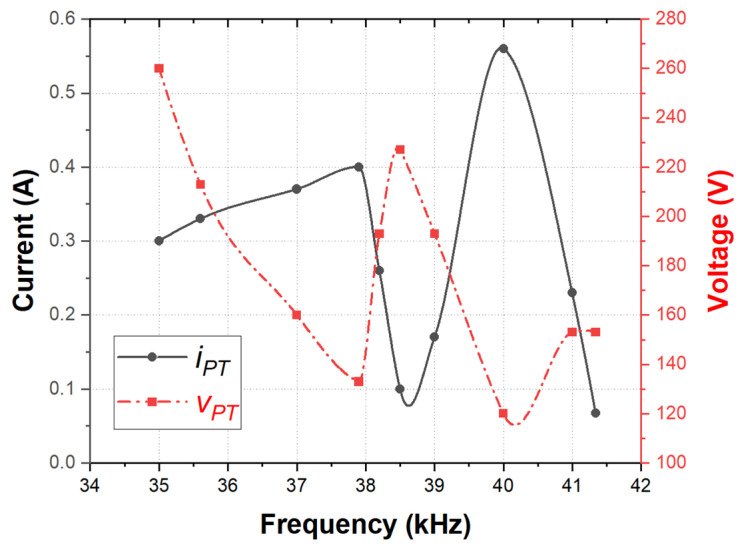
Currents ($i_{PT})$ and voltages $(v_{PT}$) in the piezoelectric transducer according to the driving frequency.

**Figure 18:**
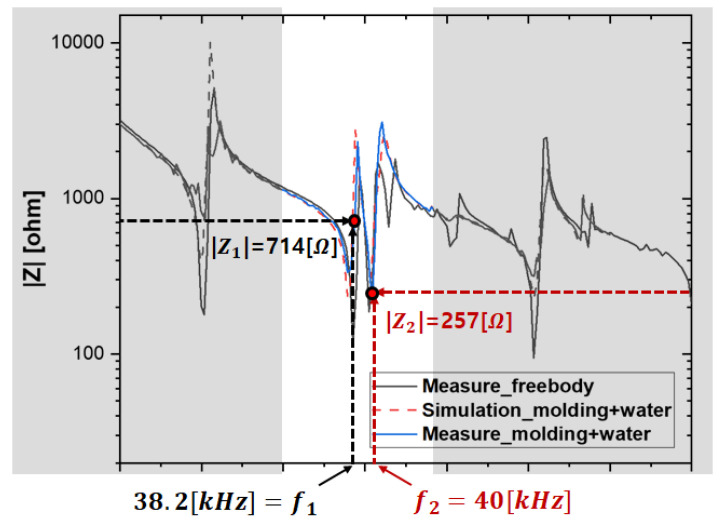
Impedance ($\left|Z\right|$) characteristics of the piezoelectric transducer under free-body and underwater conditions.

Finally, the output impedance of the piezoelectric transducer (Figure 17) was determined from the voltage ($v_{PT}$) and current ($i_{PT}$) measurements within the resonant inverter framework. The impedance represents the resistive component of the transducer at resonance. As shown in Figure 18, the impedance was verified at the optimal driving points (38.2 kHz and 40 kHz), where relatively high currents were applied, and the voltage and current were in-phase. The impedance values derived from Figure 17 are 772 Ω (@38.2 kHz, $v_{PT}$ = 193, and $i_{PT}$ = 0.25 A) and 214 Ω (@40 kHz, $v_{PT}$ = 120, and $i_{PT}$ = 0.56 A). The impedance values at the corresponding frequencies are also presented in Figure 18. The validity of the designed transducer module and the FEM characteristic analysis results under underwater load modeling for the underwater driving of the piezoelectric transducer was experimentally verified by confirming the consistency of the impedance trends obtained through the FEM characteristic analysis, the LCR meter measurements after fabrication, and the output voltage and current of the resonant VSI.

## 5. Conclusions

This study presents the design and experimental validation of an underwater ultrasonic driving system to mitigate algae blooms, which adversely affect water quality amidst the increasing severity of environmental challenges such as global warming. A Langevin-type piezoelectric transducer was used for algae removal, and its impedance characteristics were analyzed using FEM simulations. Based on these analyses, an optimal design model was proposed.

To minimize the reduction in the mechanical quality factor ($Q_{m}$) of the piezoelectric transducer, underwater environments and load conditions were modeled, and waterproof structures and sealing designs were optimized. Furthermore, an inductive compensation circuit was developed to enhance the mechanical response under underwater load conditions, enabling efficient power transfer and stable operation of the piezoelectric transducer through impedance matching.

The experimental results demonstrated that the designed transducer module exhibited mechanical responses similar to those derived from the FEM analysis. Optimal power transfer and high efficiency were achieved at the second resonance point (38.2 kHz and 40 kHz). The transducer’s mechanical performance was maximized in the series resonance mode, which is characterized by high output current and force.

This study comprehensively considered the mechanical and electrical characteristics of a piezoelectric transducer to design and optimize an underwater ultrasonic driving system. The validity of the proposed design was verified based on the FEM analysis results and experimental data. The findings of this study provide essential foundational data for the practical implementation of ultrasonic-based algae removal systems and indicate their scalability for application in various environmental and operational conditions.

## Figures and Tables

**Table 1:** Analysis results of displacement and force by housing material through FEM analysis.

Module Design Case	Resonant Frequency [kHz]	Displacement [μm]				Force [N]			
		Center	Inner	Outer	End	Center	Inner	Outer	End
Transducer + AL housing	22.4	2.47	2.49	2.57	2.54	2.3 × 10^−3^	0.62	5.85	1.22
Transducer + SUS304 housing	21.1	0.95	0.98	1.19	1.35	0.09 × 10^−3^	1.20	5.15	10.81
Transducer + Brass housing	19.9	0.7	0.74	1.03	1.29	1.2 × 10^−3^	1.79	6.51	13.83

**Table 2:** BVD-equivalent circuit parameters of the Langevin-type piezoelectric transducer.

	*C_op_*	*R*_1_	*L*_1_	*C*_1_
1st Resonance	4.40 [nF]	20.64 [Ω]	139.50 [mH]	448.83 [pF]
2nd Resonance	3.92 [nF]	40.86 [Ω]	146.28 [mH]	116.79 [pF]
3rd Resonance	3.00 [nF]	21.33 [Ω]	66.85 [mH]	102.54 [pF]

**Table 3:** Experimental results of the proposed ultrasonic driving system at 38.2 khz and 40 kHz.

	@*f_s_* = 38.2 [kHz]	@*f_s_* = 40 [kHz]
$V_{dc}$	24 [V]	24 [V]
$I_{dc}$	1.25 [A]	1.7 [A]
$P_{in}$	30 [W]	40.8 [W]
$v_{PT}\;(C1)$	193 [$V_{max}]$	120 [$V_{max}$]
$i_{PT}(C2)$	0.25 [$A_{max}$]	0.56 [$A_{max}$]

## Data Availability

Data are contained within the article.
